# Improving the Psychosocial Work Environment at Multi-Ethnic Workplaces: A Multi-Component Intervention Strategy in the Cleaning Industry

**DOI:** 10.3390/ijerph10104996

**Published:** 2013-10-14

**Authors:** Louise Hardman Smith, Kirsten Hviid, Karen Bo Frydendall, Mari-Ann Flyvholm

**Affiliations:** 1 The National Research Centre for the Working Environment, Lersø Parkallé 105, Copenhagen DK-2100, Denmark; E-Mails: lhs@nrcwe.dk (L.H.S.); kbf@nrcwe.dk (K.B.F.); 2 REMESO, Linköping University, Holmentorget 10, Norrköping SE-1601 74, Sweden; E-Mail: kirsten.hviid@liu.se

**Keywords:** intervention, psychosocial work environment, multi-ethnic workplace, cleaning

## Abstract

Global labour migration has increased in recent years and immigrant workers are often recruited into low status and low paid jobs such as cleaning. Research in a Danish context shows that immigrants working in the cleaning industry often form social networks based on shared languages and backgrounds, and that conflict between different ethnic groups may occur. This paper evaluates the impact of a multi-component intervention on the psychosocial work environment at a multi-ethnic Danish workplace in the cleaning sector. The intervention included Danish lessons, vocational training courses, and activities to improve collaboration across different groups of cleaners. Interviews about the outcome of the intervention were conducted with the cleaners and their supervisor. The Copenhagen Psychosocial Questionnaire was used as a supplement to the interviews. The results suggest that the psychosocial work environment had improved after the intervention. According to the interviews with the cleaners, the intervention had led to improved communication, trust, and collaboration. These findings are supported by the questionnaire where social support from supervisor and colleagues, social community, trust, and teamwork seem to have improved together with meaning of work, rewards, and emotional demands. The design of the intervention may provide inspiration for future psychosocial work environment interventions at multi-ethnic work places.

## 1. Introduction

Global labour migration has increased significantly over the past decade and demographics and economic interdependence suggest that migration will continue. The relationship between ethnic status and work-related health is complex [1]. However, existing data indicate that there are higher rates of occupational illnesses and injuries (including fatal injuries) among immigrants compared to native populations [2] and that immigrants are more often exposed to potentially health damaging work environments than native workers [3,4,5].

Immigrants are often recruited into low status and low paid jobs such as cleaning. In Denmark, as in other European countries, the proportion of immigrants in the cleaning industry has increased markedly in recent years [6]. In 2000, workers with immigrant background represented approximately 23% of the Danish labour force employed as traditional cleaners. This percentage increased to 35% in 2006 [7], and in larger cities the number is markedly higher. The cleaning industry faces challenges related to both the physical and the psychosocial work environment. Cleaners have an increased risk of deterioration and illnesses [8]. The psychosocial work environment is often influenced by monotonous work tasks, time pressure, changing work hours, lack of influence on jobs, lack of opportunities to develop, lack of social support, and conflicts [9,10]. Cleaners further report elevated levels of musculoskeletal pain, poor work ability, higher levels of sickness absences, and early retirement [11,12,13]. In addition, workplaces in the cleaning industry face challenges related to language proficiency and cultural diversity due to the changed composition of the workforce in recent years.

Previous research describes insufficient language proficiency among immigrant workers in the language spoken by the majority of the population as an important factor when it comes to communicating information about occupational health and safety as well as a potential factor contributing to disparity in this concern [14,15,16,17]. The diversity of languages spoken at the workplace may also be a barrier for communication and collaboration with supervisors and co-workers and may thereby affect the psychosocial work environment of immigrants as well as native workers. In a Danish context, it has been shown that immigrants working in the cleaning industry often form social networks based on shared languages and backgrounds, and that when groups of employees are tied to strong social networks based on ethnicity, conflicts between different ethnic groups may occur based on such differences [18].

In the cleaning industry, fierce competition, outsourcing, and lack of resources often hinder the introduction of initiatives aimed at improving the work environment by cleaning companies, and despite the acknowledged need for interventions only few research studies have been undertaken. Barriers to performing workplace studies among cleaners have been stated to include high employee turnover, long-term sickness absence, high proportions of immigrants, and frequent cutbacks [8,19]. Research and knowledge about which initiatives could be useful for improving the work environment is also sparse [9]. Furthermore, the studies which have been conducted have primarily focused on musculoskeletal disorders and the chemical hazards of cleaning work [6]. Only few studies have investigated the psychosocial risks [10].

In this article, we investigate possible pathways to overcome some of the challenges the multi-ethnic composition of the workforce in the cleaning industry may entail with regard to mistrust, conflicts, and poor collaboration between different groups of cleaners. This is done by evaluating the outcome of a multi-component intervention called “Make a Difference”. The intervention was designed and implemented by a municipal cleaning department to improve both the physical and psychosocial work environment of a group of cleaners working with school cleaning at a workplace with both immigrant and Danish workers.

This paper focuses on the impact of the intervention on the psychosocial work environment. Our aim is to investigate if it is possible to improve the psychosocial work environment at a multi-ethnic workplace through intervention activities which focuses on language proficiency among immigrants and interethnic interaction and collaboration, which were the main components of the intervention.

Our understanding of the psychosocial work environment is in accord with the broad definition on which the Copenhagen Psychosocial Questionnaire (COPSOQ) is based. The dimensions of the questionnaire relate to aspects such as demands at work, work organization, health and well-being, work-individual interface, interpersonal relations, leadership, and trust [20]. The COPSOQ questionnaire has been translated into several languages and has been used in several large Danish and international studies since 2000. The questionnaire has furthermore been used as a practical tool for assessing the psychosocial work environment at workplace level [20]. In comparison with the first version of the questionnaire, the second version includes scales on values at the workplace in regard to trust, justice, and social inclusiveness. The developers of the questionnaire argue that trust and justice, also referred to as social capital, have impact on recruitment and the well-being of employees as well as social processes (such as collaboration) [20]. Social capital has been linked to the quality of life at work as well as the productivity of employees [21,22,23].

As language proficiency and interethnic interaction were the focus of the intervention we primarily expected improvements in aspects related to interpersonal relations, collaboration and trust, whereas aspects related to work pace would remain unchanged since the intervention did not focus on that.

The reported study was part of a larger study carried out at workplaces with immigrant workers within the cleaning industry in Denmark. The aim was to investigate how multi-ethnic workplaces engaged in improving the work environment, and to disseminate good initiatives or best practices to others. The intention was to gain insight into the workplaces’ own opportunities and abilities to make improvements. In a second intervention, the focus was on providing courses to improve the physical work environment of the cleaners [14].

## 2. Methods

### 2.1. Description of the “Make a Difference” Intervention

The intervention was planned by the cleaning department of a Danish municipality in collaboration with consultants and had been implemented with good result at one of the other public schools in the municipality. We evaluated the impact of the intervention on the psychosocial work environment at the second school where it was carried out. The intervention was initiated in 2009 and lasted for eight months. From the cleaning department’s perspective the purpose of the intervention was to raise the standard of cleaning, improve recruitment of cleaners which had been difficult in the period of the economic upturn, and to retain employees by improving the working environment. The presumption was that this could be achieved if everybody tried to “make a difference”—for themselves as well as for others—by improving interaction and collaboration.

The intervention included work-related Danish lessons for the immigrant cleaners, and vocational training courses and a workshop on job satisfaction and teamwork for all the cleaners. Furthermore, the number of staff meetings was increased during the intervention period, and there was time scheduled for socialising in connection with most of the intervention activities. The components included in the “Make a Difference” intervention are described in more detail in Table 1.

**Table 1:** Intervention components.

Work-related Danish lessons for cleaners with immigrant background (3 h per week for 6 months) - The aim of the course was for the cleaners to be able to read the cleaning plans and talk to colleagues, supervisors, and customers about cleaning equipment, detergents, materials, and cleaning plans. Furthermore the cleaners should be able, in Danish, to call in sick and to talk about everyday life. The lessons included group work.
Vocational training courses held by an Adult Vocational Training Centre on working techniques, detergents, surfaces, and safety (8 half days) - The courses were carried out in Danish; however, instructions about safety were interpreted into relevant languages. The lessons included group work.
Workshop on job satisfaction and teamwork (2 half days) - The purpose of the workshop was to become aware of the benefits of working in a multi-cultural group of employees and to improve well-being and job satisfaction.Through exercises, games, and conversation the workshop focused on the importance of Attitudes towards work The good workday” Recognition and praise Increased community among colleagues (e.g., through role plays on the difference in being met with recognition or indifference) The workshop also included activities which were not related to work such as being taught a dance.
Staff meetings with increased frequency - The meetings focused on the implementation of the intervention and on topical topics such as how the collaboration on washing of cleaning cloths could be improved.
Social events including a summer party

All the intervention components, except for the Danish lessons, included interethnic teamwork and participation of all cleaners. Only the immigrant cleaners, who the supervisor assessed needed to improve their Danish skills, participated in the Danish lessons. Half of the Danish lessons took place during the participants’ regular work hours.

The vocational training courses were held in Danish without translation. However, groups were formed by placing employees with strong Danish language skills to assist those with poor skills. During all the courses and workshops the newly arrived immigrants, the long-term resident immigrants, and the native Danes were deliberately split-up for different types of group activities to encourage them to interact and communicate with colleagues they had not previously interacted with. All intervention components took place at the workplace. The cleaners were encouraged to bring cakes from their country of origin for coffee breaks and other forms of socialising to represent themselves to one another and to have a common starting point for conversation.

The work-related Danish lessons began before the vocational training courses, and the workshop on collaboration and job satisfaction was held before the summer party. This scheduling of courses and events was intended to give the immigrant cleaners with poor Danish skills the opportunity to improve their Danish skills, before they were to interact with colleagues during the subsequent intervention activities which were held in Danish.

Initiatives, to improve collaboration with the school on the classrooms’ readiness for cleaning, ran parallel to the other activities. The classrooms’ readiness for cleaning was the responsibility of the school and included among other things sweeping of the floors and overall tidying up by the pupils. Results on the collaboration between the cleaners and the school on these initiatives have been reported elsewhere [24].

The intervention drew on various existing initiatives and the combination of these. The work-related Danish course was a newly introduced initiative for immigrant workers which was initiated and financed partly by the Ministry of Refugee, Immigration and Integration Affairs and partly by the Ministry of Employment. The vocational training courses were part of the Adult Vocational Training programs which targets unskilled and skilled workers on the labour market and is supported the Danish Ministry of Science, Innovation and Higher Education. The National Research Centre for the Working Environment supported, through a government grant, the workshop on job satisfaction and collaboration.

### 2.2. Participants

All the cleaners employed at the school where the intervention was carried out, ten women and four men, participated in the intervention. They originated from six different countries including Romania, Thailand, Turkey, Serbia, Philippines, and Denmark.

Six of the immigrant cleaners had lived and worked in Denmark for less than two years (newcomers) and six had been in Denmark for more than ten years (long-term residents). Two cleaners were native Danes. One of the long-term residents became ill during the intervention period and therefore stopped working.

Most of the cleaners had full-time employment contracts; some, however, only worked part-time due to their health or because they had other employment. Two of the newcomers were employed 5 h a day which was the minimum hours for obtaining residency, and they shifted between different workplaces in the municipality. They hoped for more hours and a permanent workplace in the future. Some of the cleaners worked morning hours while others worked afternoon and evening shifts. They most often had a fixed area at or in the vicinity of the school to clean, such as some specific classrooms, the school dentist’s clinic, or the kindergarten which was situated on the grounds of the school. The cleaners worked in small groups or alone but most often they met up with some of their colleagues for common work tasks or breaks at some time during work hours. They also had to collaborate on tasks such as washing of cleaning cloths and disposal of waste.

### 2.3. Data Collection and Analysis

Individual semi-structured interviews [25,26,27], assisted by professional interpreters when necessary, were conducted with the cleaners at their work place three weeks before and three weeks after the intervention. An interview guide was developed which consisted of open-ended questions designed to solicit information about the cleaners’ background and their perceptions of interaction and collaboration with colleagues before the intervention as well as their expectations in regard to the intervention. After the intervention, the cleaners were asked to evaluate the different intervention components, and to tell about their experiences as well as their overall perception of the outcome of the intervention. In this article most emphasis will be put on the cleaners’ experiences and perceptions of the impact of the intervention.

We furthermore used a questionnaire based on the short version of the second version of the COPSOQ Questionnaire as a supplement to the open-ended questions during interviews to assess the psychosocial work environment at workplace level [20].

The open-ended questions in the interviews were intended to give the cleaners the possibility of expressing their personal viewpoints without preconditioned directions or limitations. The questionnaire, on the other hand, was used as an assessment tool to measure changes from before to after the intervention to the same set of questions and categories of answers.

The duration of each interview was about one hour and the interviews were conducted by the first and the second author. The cleaners were guaranteed confidentiality and participation in the interviews was voluntary. In Denmark ethical approval of studies without medical procedures is not required.

All interviews were recorded and transcribed verbatim. The transcripts were read through and index-coded using the qualitative data management program NVivo 8.0. Codes were either defined with reference to questions in the interview guide, or by themes, or novel insights that surfaced when reading the transcripts. This was followed by a content analysis of all interviews which focused on similarities and differences in experiences and perceptions among the cleaners.

Each item in the questionnaire was scored on a scale from 0 to 100 (*i.e.*, 0, 25, 50, 75, and 100 for a five response category item). The scale was computed as a mean of all items scored. In general, a score of 100 was considered most desirable. However, the opposite was true for the scales quantitative demands, work pace, emotional demands, and work-family conflict. A total of 19 scales made up the questionnaire.

In accordance with the general method used by the National Research Centre for the Working Environment for workplace surveys on the psychosocial work environment, when reporting back to (small) workplaces where statistical analysis are not justified, a 5 point difference in the mean score of before and after measurements was considered to be a marked difference in improvements or deteriorations [28,29].

In addition to interviews with the cleaners, interviews were also conducted with the supervisor before and after the intervention. The researchers were furthermore present at all types of project activities and made observations to have a common ground of reference with the cleaners during interviews and to observe whether the intervention activities were conducted as planned. The different types of qualitative and quantitative data collected were compared and similarities and differences were analyzed to assess the overall impact of the intervention on the psychosocial work environment.

## 3. Results

### 3.1. Interaction and Collaboration before the Intervention

The majority of the cleaners expressed satisfaction with the interaction and collaboration with some of their colleagues before the intervention. However many expressed limited knowledge, interaction, and collaboration with other colleagues, and some felt left out and alone. Some of the cleaners furthermore expressed dissatisfaction with the collaboration in regard to for instance washing of cloths for cleaning because they felt that only some of their colleagues did their share of this common task. A few mentioned strained relations with one particular colleague.

The newcomers primarily came from the same country of origin. They were in family with each other, and primarily interacted and collaborated within this group of cleaners. They knew very little Danish but a few had learned a little English and could communicate with the manager on behalf of the others.

Most of the long-term residents explained that they experienced good communication and collaboration with some of the other long-term residents and native Danes who had been working at the school for a long time, but that they did not communicate or interact much with the newcomers. In this group, three of the cleaners had good Danish skills, but others experienced difficulties when communicating in Danish.

Especially one of the native Danes felt left out. She missed interacting with colleagues, for example, during breaks. She regretted that neither the new nor many of the long-term resident immigrants talked much Danish. Neither she nor the other Danish cleaner spoke English or other foreign languages.

### 3.2. Expectations among the Cleaners before the Intervention

In the interviews carried out before the intervention, most of the cleaners expressed positive expectations about the intervention. This was especially the case among the newcomers who looked forward to the Danish lessons and the vocational training courses. Two of the long-term resident immigrants, who the supervisor assessed needed to improve their Danish skills, did not look forward to the Danish lessons. One of these cleaners anticipated that she would get a headache from sitting down to learn for three hours. The other cleaner was afraid to be ridiculed for his poor Danish skills by the teacher or the other cleaners. Most of the cleaners expected to improve the communication within the group of cleaners and that the standard of readiness for cleaning in the classrooms they cleaned would improve after the intervention.

### 3.3. The Cleaners’ Perceptionsof the Outcome of the Intervention

In the interviews carried out after the intervention, most of the cleaners expressed that the communication and collaboration internally in the group of cleaners had improved, but that the readiness for cleaning of classrooms had not improved much (results on the collaboration with the school about readiness for cleaning is reported elsewhere [24]). Furthermore, all the cleaners would recommend the project to cleaners at other workplaces.

Most of the cleaners expressed that improved language proficiency and increased interaction with colleagues had led to improved communication, trust, and collaboration. Further analysis of the content of the interviews showed that there were some differences in the perceptions of the outcome of the intervention among the newcomers, the long-term resident immigrants, and the native Danes. The cleaners’ perceptions of the outcome of the intervention are presented in Table 2, relative to the length of their residence in Denmark.

**Table 2:** Cleaners’ perceptions of the outcome of the intervention *.

**Newcomers**
“I have gotten a different attitude towards my work and to life in general. Improved Danish skills and improved communication has made me feel more content at work. Before, when I met other people at work, I would just say a cautious “hello”, like when passing somebody at a train station. I also communicate better with the manager for example when I have run out of products for work, and it has also become easier to have short conversations with teachers, students, and the rest of the school staff. In addition, improved knowledge of working techniques has made me more efficient and better at protecting my own body. I also learned that if you smile at the world, it will smile back and the good mood will spread and everyone will have a good day.” (Woman, 40)
“I have experienced an improvement in communication: Some colleagues I have not had difficulties communicating with in any way. However, with others it was just “hello”, “thank you”, or “goodbye”, but now we can communicate better and now we know more about each other.” (Woman, 20)
“I have improved my Danish skills and it has become easier to get to know and talk to colleagues I did not know before. When you cannot communicate with other people, both parties might get suspicious and think that the other part will not talk to you, but when you can communicate that suspicion goes away and you start to trust people.” (Man, 22)
“I have learned about cleaning agents, dosages, utensils and how to use them, and many other things. Before I thought cleaning was something everybody could do, but now I know that it is a science unto itself to clean in the right way.” (Man, 43)“After the Danish lessons we can communicate better with the pupils and the teachers on issues such as untidy classrooms and it has also become easier to talk to colleagues.” (Woman, 38)
“We really liked it. I can only recommend it.” (Woman, 22)
**Long-term residents**
“It has been absolutely fantastic for me, because I got to know the other cleaners and then you can get in contact with each other. Before there were some colleagues that I did not dare say anything to, but now I can smile at them and greet them.” (Woman, 54)“We got to know each other better, which has improved solidarity and teamwork.” (Woman, 42)
“Primarily, you get to know one another other better. However, I have taken the vocational courses before so I thought they were a waste of time for me.” (Woman, 45)
“The teamwork has improved, for example in relation to washing of cleaning cloths, and I also eat lunch with some of my colleagues now. Before I ate alone and sometimes wondered if the others were talking about me. But now I eat with the others, and I feel more confident and my Danish has also improved. It has also become easier to figure out which work tasks are mine and which tasks belong to the janitor; which tasks lies with the teacher, and which tasks the school management is responsible for.” (Man, 50)“We interact more with each other and play around a bit more and probably also take our work more seriously after the courses and the new cleaners talk more in Danish.” (Man, 52)
**Native Danes**
“We have gotten a better understanding of each other and everybody makes an effort to pay attention to each other. The Danish lessons have made it easier to communicate and work with my (immigrant) colleagues. We were encouraged to work together in groups during the courses, and therefore it is not so much them *versus* us as it was before, because before they were all new and they all spoke the same language. Now we interact more with each other. I do not know the cost of the intervention, but in my opinion it has been worth every penny.” (Woman, 52)
“The newcomers have learned to clean better, but I think it has been a bit of a waste of time for those who have taken the vocational course before. The Danish lessons for the immigrants have, however, made it easier for me to communicate with my colleagues.” (Woman, 59)

* The presented perceptions are condensed excerpts from the interviews.

The *newcomers* especially emphasized the experience of trusting colleagues more after the intervention. One of the newcomers described how she, before the intervention, experienced the workplace as a train station (an impersonal place). In her experience, the intervention had led to a sense of belonging and improved job satisfaction. The same sense of belonging to the workplace or a team also applied to other newcomers after the intervention. Another of the newcomers explained that the intervention had resulted in improved Danish skills and an opportunity to be together with other people and to get to know colleagues she did not know beforehand.

Some of the *long-term resident* immigrant cleaners were of the perception that the intervention had benefitted the newcomers the most. This was especially the case for the courses on working techniques which they perceived were unnecessary for them as they had taken these courses before. Similar to the newcomers, the long-term residents, however, also emphasized better knowledge of each other and improved trust, teamwork, and collaboration within the group of cleaners as an outcome of the intervention. One of the men especially emphasized the importance of the group work in connection with the different intervention activities. In his opinion, the group work had resulted in better collaboration in the group of cleaners after the intervention. The two long-term resident cleaners who were sceptical about the Danish lessons before the intervention had both decided to attend the first lessons to give the course a try, and both had continued throughout the six month duration of the course. The person who felt his Danish skills were very poor actually experienced ending up helping the newcomers with their Danish skills during the lessons. He explained that he, due to this, had experienced improved relationships with this group of colleagues.

The *native Danes* regretted the lack of collegiality at the workplace before the intervention. They were not proficient in English or other of the languages spoken by the immigrants, and as such they had become a Danish speaking minority at the workplace. They especially highlighted the Danish lessons for the immigrant cleaners, which in their perception had improved the communication and teamwork with their colleagues the most. They were, however, also critical of the courses on working techniques like the long-term residents, and for the same reasons.

### 3.4. Changes in the Psychosocial Work Environment according to the COPSOQ Questionnaire

According to the COPSOQ questionnaire, the cleaners rated their overall psychosocial work environment to be surprisingly good before the intervention, compared to the average Danish wage-earner [30].

**Figure 1:**
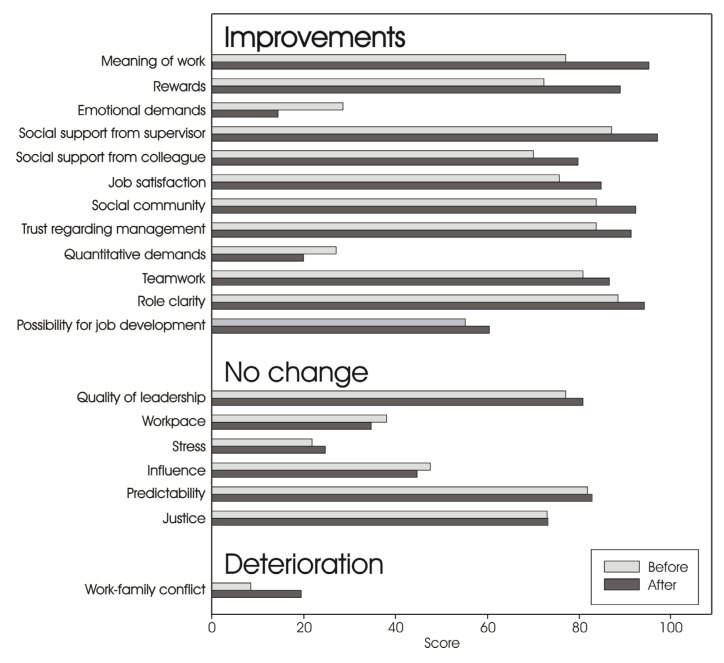
Changes in the psychosocial work environment.

However, according to the 5 point method used, a positive difference from before to after the intervention had occurred in 12 of the 19 scales, six scales remained unchanged, and one had deteriorated. The 19 scales from the COPSOQ questionnaire are depicted in Figure 1 in descending order, starting with the scale showing the most marked improvement from before till after the intervention.

Of the scales from the COPSOC questionnaire positive changes were observed in regard to scales such as meaning of work, perceived rewards, level of emotional demands, social support from supervisor and colleagues as well as job satisfaction, social community, and trust regarding management. Presumably due to more energy spent on learning Danish there was an increase in Work-family conflict. However, it did not affect the stress level of the participants.

### 3.5. The Supervisor’s Perception of the Outcome of the Intervention

The supervisor was satisfied with the outcome of the intervention. In her experience the immigrant cleaners had obtained better Danish skills and her communication with the group of cleaners had improved. The supervisor had furthermore experienced improvements in communication and collaboration internally among the cleaners, as they were now capable of coordinating tasks, such as washing of cleaning cloths and disposal of waste, without her assistance, something which previously had been impossible.

## 4. Discussion

The intervention gave the group of cleaners time to learn and practice new skills, and to interact and collaborate with each other across different ethnic groups, all of which seemed to have improved the psychosocial work environment. In the interviews, the cleaners especially emphasised aspects such as improved language proficiency, interaction, communication, and trust and cooperation in relation to colleagues as the outcome of the intervention. Similar improvements could also be found in the answers from the questionnaires, as scales such as social support from supervisor, social support from colleagues, social community, trust, and teamwork had improved from before to after the intervention together with meaning of work, rewards, and emotional demands.

Aspects such as cooperation and trust, which have both been associated with the concept of social capital, were highlighted as improved by the cleaners in both the interviews and the questionnaires [21,22,23].

The combination and scheduling of the different components in a multi-component intervention is important. In this regard the current intervention seems to have been well planned. The combination of work-related Danish lessons with vocational training courses and workshops on collaboration as well as increased frequency of staff meetings made it possible for the cleaners to communicate and interact with each other. That the intervention began with the Danish lessons made it possible for the cleaners to communicate during the following intervention activities. The placement of the participants in groups of colleagues they had not previously interacted with, during the courses and workshops, furthermore contributed to the improvement in communication and collaboration. Thus, the combination and scheduling of the different components seem to have enhanced each component, which would not have been as successful in their own.

The intervention also focused on improving the social community of the group of cleaners through a summer party and through a workshop on job satisfaction and communication which included non-job related activities. The activities were appreciated and perceived as recognition of their work by the cleaners, who were not accustomed to such initiatives at their present or former workplaces.

The multi-component design of the intervention, including work-related language lessons as well as vocational training and initiatives to improve collaboration, makes it a unique work environment intervention in the cleaning trade. Most often interventions include only one or a few components and only address few work environment factors [9]. This study adds to the growing evidence that multi-component interventions have a greater chance of success than single component interventions [31]. The design of the intervention may inspire future research with important insights on how to design interventions which aim to improve the psychosocial work environment at multi-ethnic workplaces. However, the design cannot be transferred indiscriminately to other multi-ethnic workplaces in other sectors, nor may it be useful for all multi-ethnic cleaning companies. School cleaning is a special type of cleaning where several cleaners work at the same time at the same workplace. Office cleaning is often organized in a similar manner, whereas cleaning in small companies or private households is most likely done by cleaners working alone without colleagues. 

Both the native Danish cleaners and the long-term resident immigrant cleaners were critical towards the courses on working techniques as they had previously participated in similar courses. In future interventions more emphasis should therefore be put on explaining why it is important that everyone participates and also to give those who have attended before well-defined tasks such as assisting the newly arrived cleaners during the courses.

The overall good ratings of the psychosocial work environment before the intervention according to the COPSOQ questionnaire may in part be explained by the immigrant cleaners’ low expectations. Their previous work experiences included poor working conditions in their countries of origin and/or longer periods of unemployment.

In addition, a Danish study has shown that non-Western cleaners compared to native Danish cleaners reported significantly higher on a number of scales from the COPSOC questionnaire [32]. This may in part explain why the cleaners in the current study rated the psychosocial work environment surprisingly high as the majority of the cleaners had immigrant backgrounds.

To maintain the improvements in language skills, communication, collaboration, and trust achieved during the “Make a Difference” intervention, continuation of activities and events where all the cleaners can get together are important. In a geopolitical context, where tensions exist between different ethnic and cultural groups, management should furthermore focus on how to avoid possible conflicts deriving from such tensions.

In future interventions aiming at improving the psychosocial work environment in the cleaning sector for immigrant as well as native cleaners, initiatives at the organizational or structural level would be relevant. The initiatives should address factors such as lack of opportunities to develop, changing work hours, and insecure job situations which have not been in focus in this intervention.

## 5. Conclusions

The findings suggest that the psychosocial work environment at multi-ethnic workplaces can be improved by applying multi-component interventions focusing on language proficiency, interethnic communication and collaboration as well as by introducing social events. The latter may especially be the case in occupations, were such initiatives are rare as is the case in cleaning. According to the interviews with the cleaners, the intervention had led to improved communication, trust, and collaboration. These findings are supported by the questionnaire where social support from supervisor and colleagues, social community, trust, and teamwork seem to have improved together with meaning of work, rewards, and emotional demands. The design of the intervention may provide inspiration for future psychosocial work environment interventions at multi-ethnic work places.
